# Complete mitochondrial genome of *Pseudocentema liui* (Phasmatodea; Lonchodidae）

**DOI:** 10.1128/mra.00622-25

**Published:** 2025-08-11

**Authors:** Ting Luo, Haiyu Luo, Jing Guo, Xun Bian

**Affiliations:** 1 Key Laboratory of Ecology of Rare and Endangered Species and Environmental Protection, Guangxi Normal University, Ministry of Education 12388, Guilin, China; University of Maryland School of Medicine, Baltimore, Maryland, USA

**Keywords:** mitochondrial genome, Necrosciinae, Hainan

## Abstract

Here, we present the complete mitochondrial genome (16,250 bp) of *Pseudocentema liui*, Hainan stick insect, characterized by a circular double-stranded structure containing 13 protein-coding genes, 22 transfer RNAs, 2 ribosomal RNAs, and a control region (D-loop).

## ANNOUNCEMENT

*Pseudocentema* Chen, He & Li, 2002 is endemic to Hainan, China, and currently comprises two known species, namely, *Pseudocentema bispinatum* Chen & He, 2002 and *Pseudocentema liui* Ho, 2013 (1, 2). *P. liui* is mainly distributed in Bawangling National Nature Reserve, Diaoluoshan National Forest Park, Yinggeling Nature Reserve, and Wuzhishan National Nature Reserve (2–4). Now, the species focused on the morphological description without the mitogenome reported (5). This study sequenced and annotated the complete mitochondrial genome of *P. liui*.

One specimen of *P. liui* was collected from Qiongzhong, Hainan (19.155°N, 110.332°E), preserved in 100% ethanol at Guangxi Normal University. Genomic DNA was extracted using the TIANamp Genomic DNA Kit (TIANGEN, Beijing, China) from hind leg muscle tissue and sequenced on the DNBSEQ sequencing platform (Shenzhen BGI Genomics Co., Ltd.) with 150 bp paired-end reads. The raw sequencing data were processed using SOAPnuke for adapter trimming and quality filtering (6). Reads containing ≥50% bases with quality scores <10 were discarded, yielding 20,004,183 high-quality clean reads. The mitochondrial genome (mitogenome) was assembled using NOVOPlasty v.4.2.1 with the following parameters: assembly type = mito, genome range = 14,000–18,000, k-mer = 39, max memory = 16, extended log = 0 (7), which selected *Neohirasea stephanus* OL405132 (8) as reference using CLC Genomics Workbench 12 with default parameters (9). The mitogenome was initially annotated using the MITOS2 webserver on the Galaxy platform data set with the fifth invertebrate genetic code, the RefSeq 89 Metazoa as reference data (10), and manually checked by MEGA v.11, followed by start/stop codon verification (11). The circular map was drawn with OrganellarGenomeDRAW v.1.3.1 (12).

**Fig 1:**
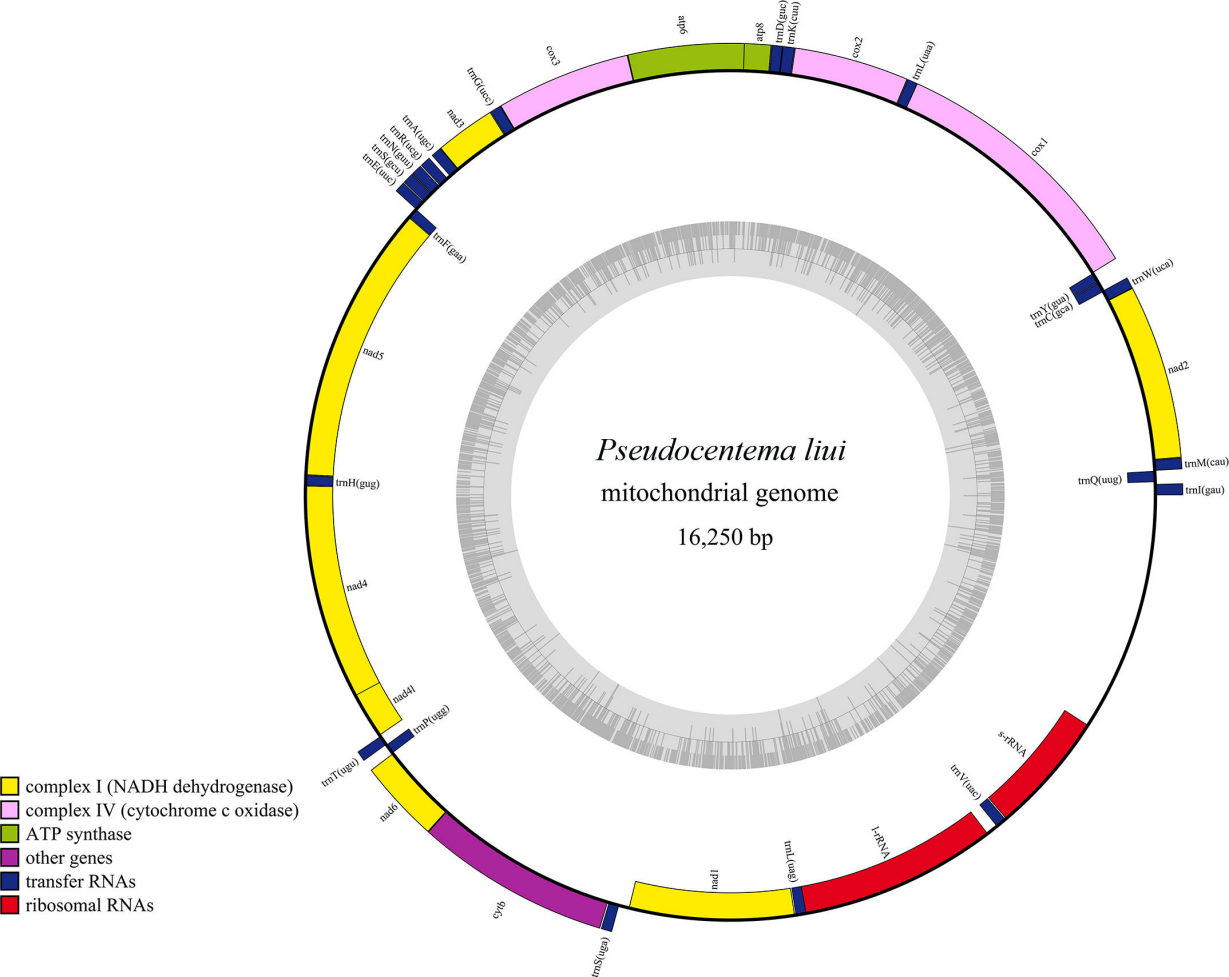
Complete mitogenome map of *Pseudocentema liui* in this study. The innermost gray-colored ring displays GC content distribution. Genes transcribed clockwise are positioned on the outside, while those transcribed counterclockwise are located on the inside. The color coding denotes distinct gene groups, as specified in the key located in the bottom left corner.

The complete mitogenome of *P. liui* (GenBank under accession number PV700088) was assembled into a circular structure of 16,250 bp in length, with an A + T content of 77%. The mitogenome contained 13 protein-coding genes, 22 transfer RNAs (tRNAs), two ribosomal RNAs (12S rRNA and 16S rRNA), and a control region, as illustrated in Fig. 1. As shown in Table 1, the heavy strand encodes 23 genes, compared to 14 genes encoded by the light strand. Among the protein-coding genes, start codon usage was as follows: ATG for cox1, atp6, cox3, nad4, nad4L, and cob (six genes); ATA for cox2, nad3, nad6, and nad1 (four genes); ATT for nad2 and nad5 (two genes); and ATC for atp8 (one gene). The TAA termination codon was used in all protein-coding genes, except for nad3 and cob, which used TAG, and cox2 and nad5, which used T-., as the termination codon in animal mitochondrial mRNAs is formed through 3′ polyadenylation, compensating for incomplete stop codons encoded in the genome (13, 14). The tRNA genes comprise 22 sequences, with lengths ranging from 64 bp (trnC, trnL2, trnH, trnT, and trnP) to 70 bp (trnK and trnV). The rRNA genes include rrnS (762 bp) and rrnL (1,235 bp).

**TABLE 1:** Mitochondrial genome architecture, gene content, and codon usage in *P. liui*

Gene	Location	H/L strand^*a*^	Gene length (bp)	Intergenic region length (bp)	Start/stop codons
trnI	1–69	+	69	11	
trnQ	81–149	−	69	0	
trnM	150–216	+	67	0	
nad2	217–1233	+	1,017	−2	ATT/TAA
trnW	1232–1300	+	69	−8	
trnC	1293–1356	−	64	0	
trnY	1357–1423	−	67	1	
cox1	1425–2963	+	1,539	−5	ATG/TAA
trnL2	2964–3022	+	64	0	
cox2	3023–3692	+	670	0	ATA/T^*b*^
trnK	3693–3762	+	70	−1	
trnD	3762–3826	+	65	0	
atp8	3827–3985	+	159	−7	ATC/TAA
atp6	3979–4656	+	678	−1	ATG/TAA
cox3	4656–5441	+	786	−1	ATG/TAA
trnG	5441–5508	+	68	0	
nad3	5509–5862	+	354	−2	ATA/TAG
trnA	5861–5926	+	66	19	
trnR	5946–6010	+	65	3	
trnN	6014–6080	+	67	0	
trnS1	6081–6148	+	68	1	
trnE	6150–6215	+	66	−2	
trnF	6214–6278	−	65	0	
nad5	6279–8001	−	1,723	0	ATT/T^*b*^
trnH	8002–8065	−	64	0	
nad4	8066–9397	−	1,332	−7	ATG/TAA
nad4l	9391–9678	−	288	5	ATG/TAA
trnT	9684–9747	+	64	0	
trnP	9748–9811	−	64	7	
nad6	9813–10295	+	483	−1	ATA/TAA
cob	10295–11431	+	1,137	5	ATG/TAG
trnS2	11437–11502	+	66	60	
nad1	11563–12582	−	1,020	111	ATA/TAA
trnL1	12586–12653	−	68	−19	
rrnL-b	12654–13869	−	1,235	69	
trnV	13939–14008	−	70	3	
rrnS	14012–14773	−	762		

^*a*^ +, heavy (H) strand; −, light (L) strand.

^*b*^ Truncated termination codon.

## Data Availability

The complete mitochondrial genome sequence of *Pseudocentema liui* is available in GenBank under accession number PV700088. The associated BioProject, SRA, and BioSample numbers are PRJNA1244121, SRR33562310, and SAMN47731654, respectively.
